# Band structural and absorption characteristics of antimonene/bismuthene monolayer heterojunction calculated by first-principles

**DOI:** 10.3389/fchem.2022.973516

**Published:** 2022-08-05

**Authors:** Yanyan Zhan, Xuan Fang, Dengkui Wang, Dan Fang, Bobo Li, Jinhua Li, Xiaohua Wang

**Affiliations:** ^1^ State Key Laboratory of High Power Semiconductor Lasers, School of Science, Changchun University of Science and Technology, Changchun, China; ^2^ School of Science and Engineering, The Chinese University of Hong Kong, Shenzhen, China; ^3^ College of New Materials and New Energies, Shenzhen Technology University, Shenzhen, China

**Keywords:** antimonene (Sb), bismuthene (Bi), lateral heterojunction, band structure, optical absorption, first-principles calculations

## Abstract

The band gap of lateral heterojunctions (LHSs) can be continuously tuned by changing the widths of their components. In this work, Sb/Bi LHSs based on monolayer Sb and Bi atoms with armchair and zigzag interfaces are constructed, respectively. It exhibits an atom’s number in planner-dependent tunable band gap and near-infrared range absorption characteristics. They are systematically studied by first-principles calculations. The widths are represented by the number (n) of Sb or Bi atom chains. When n increases from 2 to 8, the bandgaps of armchair Sb_n_/Bi_n_ LHSs decrease from 0.89 to 0.67 eV, and the band gaps of zigzag Sb_n_/Bi_n_ LHSs decrease from 0.92 to 0.76 eV. The partial density of states spectra indicate that the occupied states of the valence band are mainly provided by the Bi 6*p* orbitals. Additionally, the unoccupied states of the conduction band are always provided by the Sb 5*p* orbitals and Bi 6*p* orbitals. For Sb_n_/Bi_n_ LHSs, the absorption edge along XX and YY directions move toward the long wavelength direction. These results provide an approach for the applications of two-dimensional materials in near-infrared devices.

## Introduction

The influence of quantum confinement effect on the band structure of semiconductor cannot be neglected, especially for tuning of the band gap (Huang et al., 2020; Mu et al., 2018; Agafonov et al., 2010). It is well known that the band gap is caused by quantum confinement effect due to the size. As the size increases, and the band gap decreases (Wu et al., 2005; Wilcoxon et al., 1997). Researchers have used quantum confinement to adjust the band structures of semiconductor materials (Arutyunov et al., 2018; Peng et al., 2018; Chang et al., 2012; Jarolimek et al., 2014). For example, the quantum well is a significant material with a discrete energy level. Its band gaps always decrease as the quantum well thickness increases (Jarolimek et al., 2014). This research indicates that the size of the semiconductor is crucial in adjusting the electronic properties.

Two-dimensional (2D) materials have been intensely studied because of their electronic and optical properties, which can be widely used in optoelectronics, MOSFET, and various devices (Zhang et al., 2021; Das et al., 2015; Shi et al., 2010; Glavin et al., 2020; Zhang et al., 2018; Sturala et al., 2019; Liu et al., 2022; Kumar and Ahluwalia, 2013). It exhibits the numbers of layers dependent on tunable band gaps and variable band structure. For instance, the single-layer MoS_2_ is a direct band semiconductor, but the bulk MoS_2_ shows indirect band characteristics (Singh et al., 2012). These phenomena are attributed to the interaction of electrons between layers. Because the layers are connected by Van der Waals forces rather than chemical bonds, the interaction is always weak. The interfaces between layers also increase the electron scattering, which greatly limits the performance of layered materials. But, in the LHSs, the atoms are connected by chemical bonds, which ensures strong electron interaction. A sea of research results show that the electronic properties of 2D materials can be improved with the construction of LHSs (Son et al., 2016; Feng et al., 2021; Guo et al., 2020; Du et al., 2007). However, the size of each component in LHSs has a great influence on the electron interaction due to the limitation of action distance (Zhang et al., 2021; Li et al., 2021; Lee et al., 2017; Bar Yoseph et al., 2014). Therefore, it is meaningful to investigate the band properties of LHSs constructed by various sizes.

In this article, two kinds of single-layer Sb/Bi LHSs composed of monolayer Sb and monolayer Bi with various widths are designed. The widths are represented by n of Sb or Bi atom chains. Initially, the stabilities of Sb/Bi LHSs are proved by formation energies. Then, the band structure, density of states (DOS), and absorption of Sb/Bi LHSs are analyzed in detail by first-principles calculations. It is easy to find that all Sb/Bi LHSs exhibit the feature of direct band structure. Their band gaps are also reduced with an increase in n. For the two types of LHSs, Sb_n_/Bi_n_ LHSs, the occupied states of valence band are mainly provided by the Bi 6*p* orbitals. Moreover, the unoccupied states of the conduction band are always provided by the Sb 5*p* orbitals and Bi 6*p* orbitals. The effect of n on the absorption characteristics of Sb/Bi LHSs is also studied. When n increases, the absorption intensity along the XX direction and the YY direction decreases. Undoubtedly, these Sb/Bi LHSs have potential applications in the field of near-infrared optoelectronic devices.

## Computational details

In this article, the first-principles calculations based on the density functional theory (DFT) were performed in the Vienna ab initio simulations package (VASP) (Kresse and Hafner, 1993; Kresse and Furthmüller, 1996). The electron exchange-functional is treated by the Perdew–Burke–Ernzerhof (PBE) functional (Perdew et al., 1992) and described by the generalized gradient approximation (GGA) (Stewart et al., 2005). A cutoff energy of 600 eV was chosen for the plane-wave expansion of wave functions, and the Monkhorst–Pack scheme of k-point sampling was adopted for the integration over the first Brillouin zone. A 2 × 9 × 1k-mesh was used during structural optimization until the energy and Hellman–Feynman force are converged within 10^–6^ eV and 0.01 eV/Å, respectively (Souadi et al., 2017). In order to minimize the artificial interaction between layers and their periodic images, a vacuum slab of 15 Å along the *z*-axis was introduced. The electronic band structure, the density of states (DOS), the valence band maximum/the conduction band minimum (VBM/CBM), and the optical absorption spectra of each 2D LHS were calculated by PBE.

## Results and discussion

Generally, the properties of LHSs are determined by the nature of the building blocks. Therefore, the LHSs with suitable constituent materials can exhibit unique properties and have a broad application field. In this work, monolayer Sb and monolayer Bi are connected by covalent bonds to form Sb_n_/Bi_n_ LHSs. They have the same hexagonal structures (Bian et al., 2016; Ji et al., 2016; Wu et al., 2017; Tian et al., 2021). For this reason, the sharp hetero-interface and intact hexagonal structures are ensured. Considering the two types of edges, armchair and zigzag, in hexagonal structures, Sb_n_/Bi_n_ LHSs are constructed along the armchair edge and zigzag edge, respectively. The Sb and Bi atom chains alternately appear in the direction perpendicular to the interface. Supplementary Figure S1 schematically illustrates the arrangements of Sb_n_/Bi_n_ LHSs. In Supplementary Figure S1E, their Brillouin zone presents a rectangular structure. The Γ-point is the position of the maximal symmetry point.

High stability is the prerequisite for the construction of LHSs; therefore, the formation energies of all Sb_n_/Bi_n_ LHSs are calculated to first evaluate their stability. As shown in Supplementary Figure S2, the formation energies of all LHSs are negative, which indicates that these LHSs are stable. For armchair structure Sb_n_/Bi_n_ LHSs, the formation energies decreased from −2.498 eV to −2.506 eV when the n is further increased. Moreover, the formation energies of zigzag Sb_n_/Bi_n_ LHSs decreased from −2.498 eV to −2.526 eV. For the two types of Sb_n_/Bi_n_ LHSs, formation energies decrease when n increases from 2 to 8. The calculation results indicated that the constructed LHSs based on Sb_n_/Bi_n_ would be achieved, regardless of the interface atom arrangement.

**FIGURE 1:**
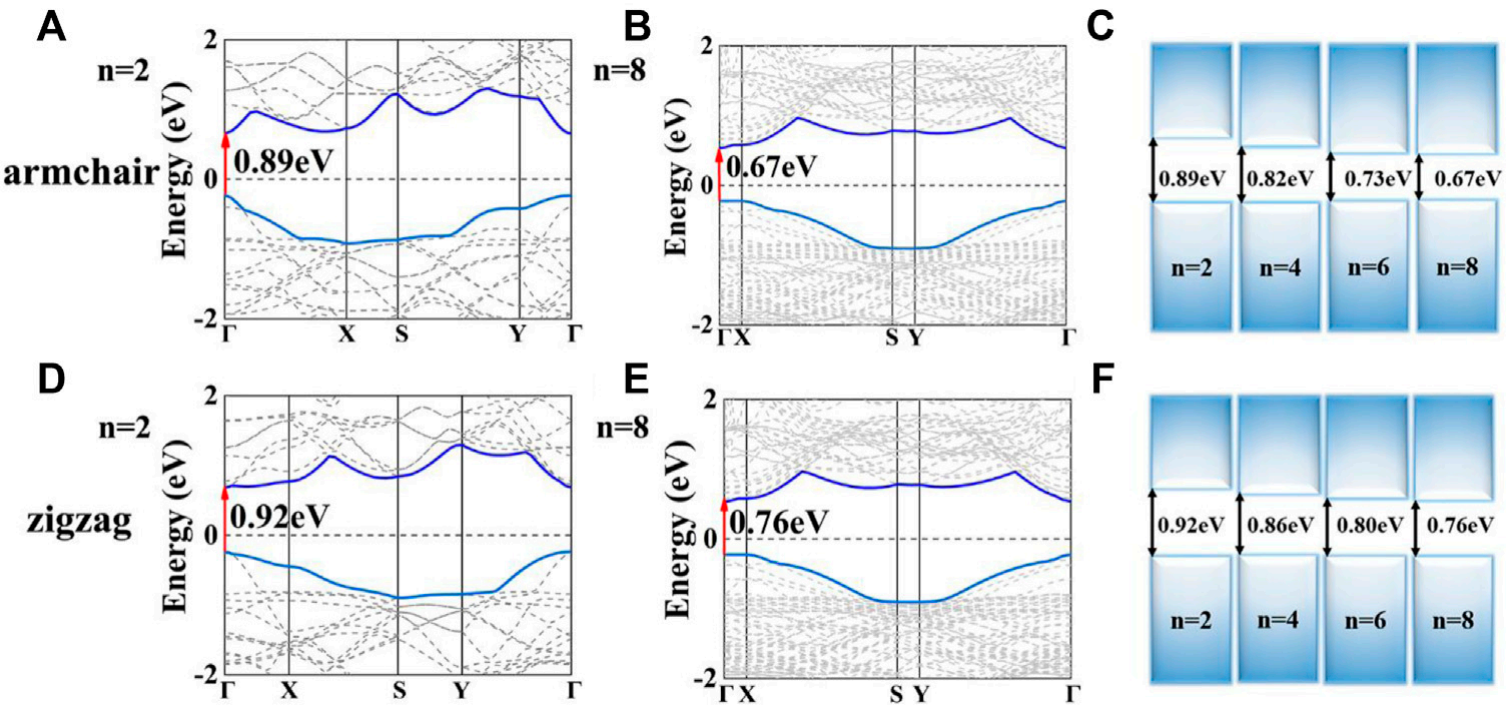
**(A,B)** Band structure of the armchair of Sb_2_/Bi_2_ LHSs and Sb_8_/Bi_8_ LHSs, respectively; **(C)** Band gap of the armchair Sb/Bi LHSs under n from 2 to 8; respectively. **(D,E)** Band structure of the zigzag Sb_2_/Bi_2_ LHSs and Sb_8_/Bi_8_ LHSs, respectively; **(F)** Band gap of the zigzag Sb/Bi LHS under n from 2 to 8, respectively.

Based on the stable structures of Sb_n_/Bi_n_ LHSs, we now discuss their electronic properties. The band structures of Sb_n_/Bi_n_ LHSs with armchair and zigzag interfaces are illustrated in Figure 1, Supplementary Figure S3. For armchair structure Sb_n_/Bi_n_ LHSs, they all exhibit direct gap characteristics at the Γ-point. In Figure 1C, their band gaps decrease from 0.89 to 0.67 eV when n is from 2 to 8. Furthermore, the difference between the band gaps of Sb_6_/Bi_6_ and that of Sb_8_/Bi_8_ is 0.06 eV. It is less than the band gap difference between Sb_2_/Bi_2_ and Sb_4_/Bi_4_. Considering both effects, the band gaps of Sb_n_/Bi_n_ LHSs decrease when n is further increased. Similarly, the band structures of the zigzag structure Sb_n_/Bi_n_ LHSs are same as those of armchair Sb_n_/Bi_n_ LHSs, except for the value of band gaps. Its band gaps decrease to 0.76 eV as presented in Figure 1F.

Supplementary Figure S4 shows the total density of states (TDOS) and partial density of states (PDOS) are studied. For armchair structure Sb_n_/Bi_n_ LHSs, the main occupied state of the valence band of Sb_2_/Bi_2_ LHS is provided by the Sb 5*p* orbital and Bi 6*p* orbital. Nevertheless, the occupied states of the valence band are mainly provided by the Bi 6*p* orbitals with the increase in n. In contrast, the unoccupied states of the conduction band are always provided by the Sb 5*p* orbitals and Bi 6*p* orbitals. Moreover, the valence band is always close to the Fermi level under n from 2 to 8, which indicates that Sb and Bi atoms form covalent bonds at interfaces. However, there is little shift in the valence band. This further leads to the strong direct interaction of the Sb and Bi atoms through covalent bonds at interfaces. Moreover, the conduction band shifts negatively. This is the direct reason for the reduction in the band gap of Sb_n_/Bi_n_ LHSs. The zigzag structure Sb_n_/Bi_n_ LHSs shows consistent electronic properties.

Next, the band-decomposed partial charge densities for VBM and CBM of two types of LHS are presented in Supplementary Figure S5. For armchair structure Sb_n_/Bi_n_ LHSs, the charges on VBM of Sb_2_/Bi_2_ LHS are distributed in an average manner around Sb atoms and Bi atoms in Supplementary Figure S5A_1_. Supplementary Figure S5A_2_ presents that the charges on VBM of Sb_4_/Bi_4_ LHS are distributed at Bi–Bi bonds and the interface. Moreover, the charges on VBM are all distributed at the Bi–Bi bonds when *n* = 6 and *n* = 8. It disappears at the interface between Sb and Bi. Evidently, the charges on VBM gradually transfer to the Bi–Bi bonds. It indicates that the Sb_n_/Bi_n_ LHSs have good electronic properties (Tian et al., 2021). For CBM, the charges of Sb_2_/Bi_2_ LHS and Sb_4_/Bi_4_ LHS are averagely distributed on Sb atoms and Bi atoms, as illustrated in Supplementary Figures S5B_1_, B_2_. However, the charges of Sb_6_/Bi_6_ LHS and of Sb_8_/Bi_8_ LHS are distributed on Bi atoms and at the interface. In addition, the charges on VBM of zigzag structure Sb_n_/Bi_n_ LHSs show a phenomenon similar with that of armchair structure. The only difference between them is that the charges on VBM show localization on the Bi–Bi bonds for the zigzag structure, as shown in Supplementary Figures S5C_2_–C_4_. For CBM of zigzag structure Sb_n_/Bi_n_ LHSs, the charges are averagely distributed on Sb atoms and Bi atoms, regardless of the value of n.

**FIGURE 2:**
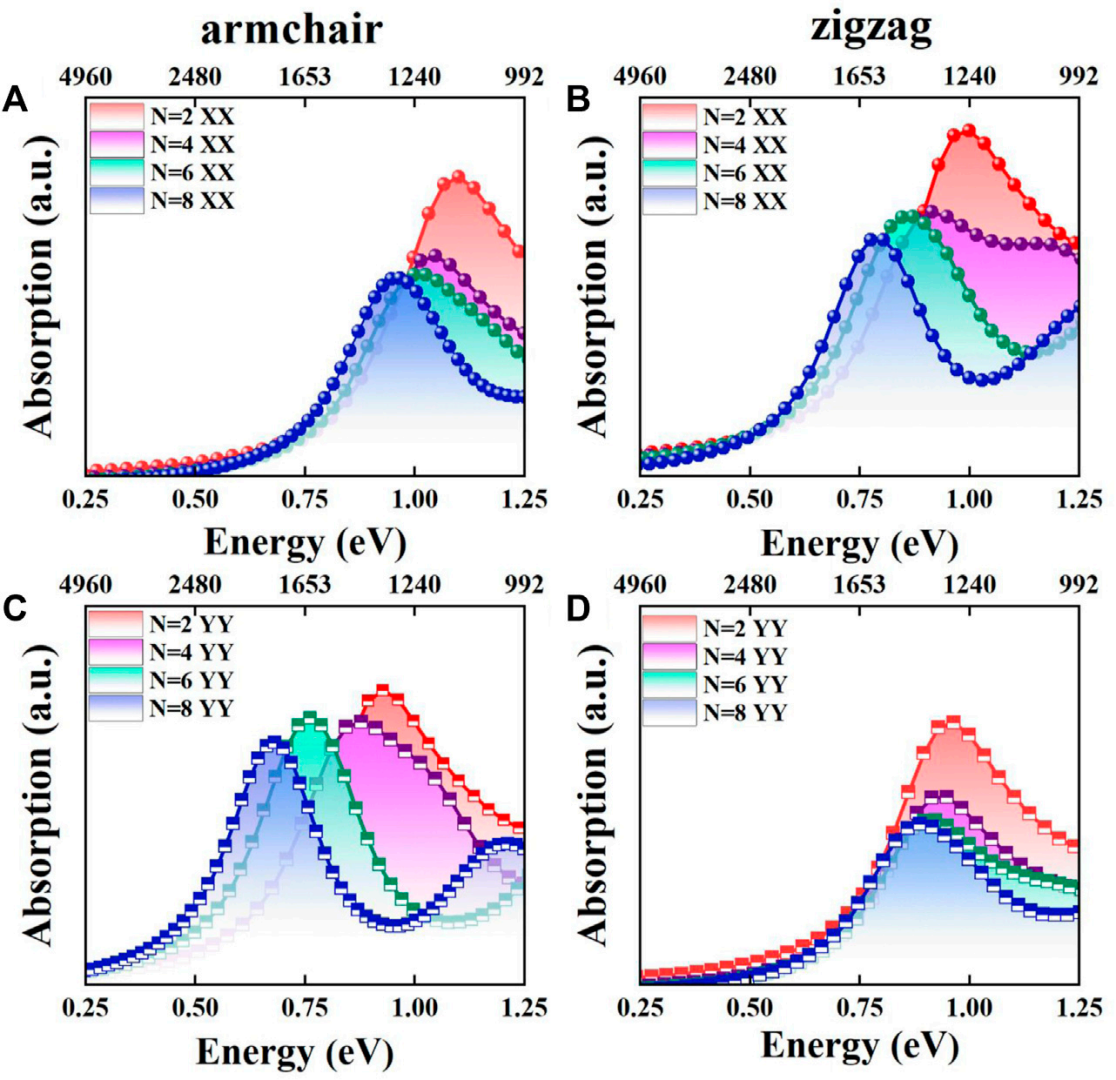
**(A,C)** Absorption spectra of the armchair Sb_n_/Bi_n_ LHSs in XX and YY directions under n from 2 to 8, respectively; **(B,D)** Absorption spectra of the zigzag Sb_n_/Bi_n_ LHSs in XX and YY directions under n from 2 to 8, respectively.

Shown in Figure 2 are the optical absorption spectra of the Sb_n_/Bi_n_ LHSs. For armchair structure Sb_n_/Bi_n_ LHSs, the absorption edges along XX and YY directions are located in the near-infrared range. Furthermore, the absorption edges move toward the long wavelength direction. In this context, the absorption intensity is reduced under n from 2 to 8. The absorption spectrum is found to be consistent with the band gap distribution. Furthermore, the absorption in the XX direction of the Sb_n_/Bi_n_ LHSs is stronger than that in the YY direction. It is reported that the application direction of band gap engineering is related to the carrier transmission direction (Li et al., 2021). When the Sb_n_/Bi_n_ LHSs are designed, the XX direction corresponds to the interface between the Sb and Bi units. Therefore, the transfer of carriers along the XX direction has the greatest possibility. The zigzag structure Sb_n_/Bi_n_ LHSs shows consistent optical absorption properties. The results show that Sb_n_/Bi_n_ LHSs have applications in near-infrared devices.

## Conclusion

In conclusion, the band structure, electronic, and optical absorption properties of the Sb/Bi LHSs are determined by the first-principles calculations. Our investigations show that these LHSs are theoretically stable and possible to construct. The structure also becomes more stable with the increasing n. The results show that both armchair and zigzag Sb/Bi LHSs exhibit adjustable direct band gap. Moreover, the band gaps decrease with an increase in n. Then, the PDOS and the VBM/CBM are used to specify the electronic state of Sb_n_/Bi_n_ LHSs. For the two types of LHS, the occupied states of the valence band are provided by the Bi 6*p* orbitals. Moreover, the unoccupied states of the conduction band are always provided by the Sb 5*p* orbitals and Bi 6*p* orbitals. In addition, Sb/Bi LHSs exhibit near-infrared range absorption characteristics. The absorption XX and YY edges move toward the long wavelength direction. Furthermore, it shows that the construction of these LHSs will promote the application of VA group elements in near-infrared optoelectronic devices in the future.

## Data Availability

The raw data supporting the conclusion of this article will be made available by the authors, without undue reservation.
