# Supplementary material for: Band structural and absorption characteristics of antimonene/bismuthene monolayer heterojunction calculated by first-principles
Source: Front Chem. 2022 Aug 5;10:973516. doi: 10.3389/fchem.2022.973516 (PMC9388903; doi:10.3389/fchem.2022.973516)
Supplement: Supplementary file 1 [file DataSheet1.docx]

Supplementary Material

## Supplementary Figures


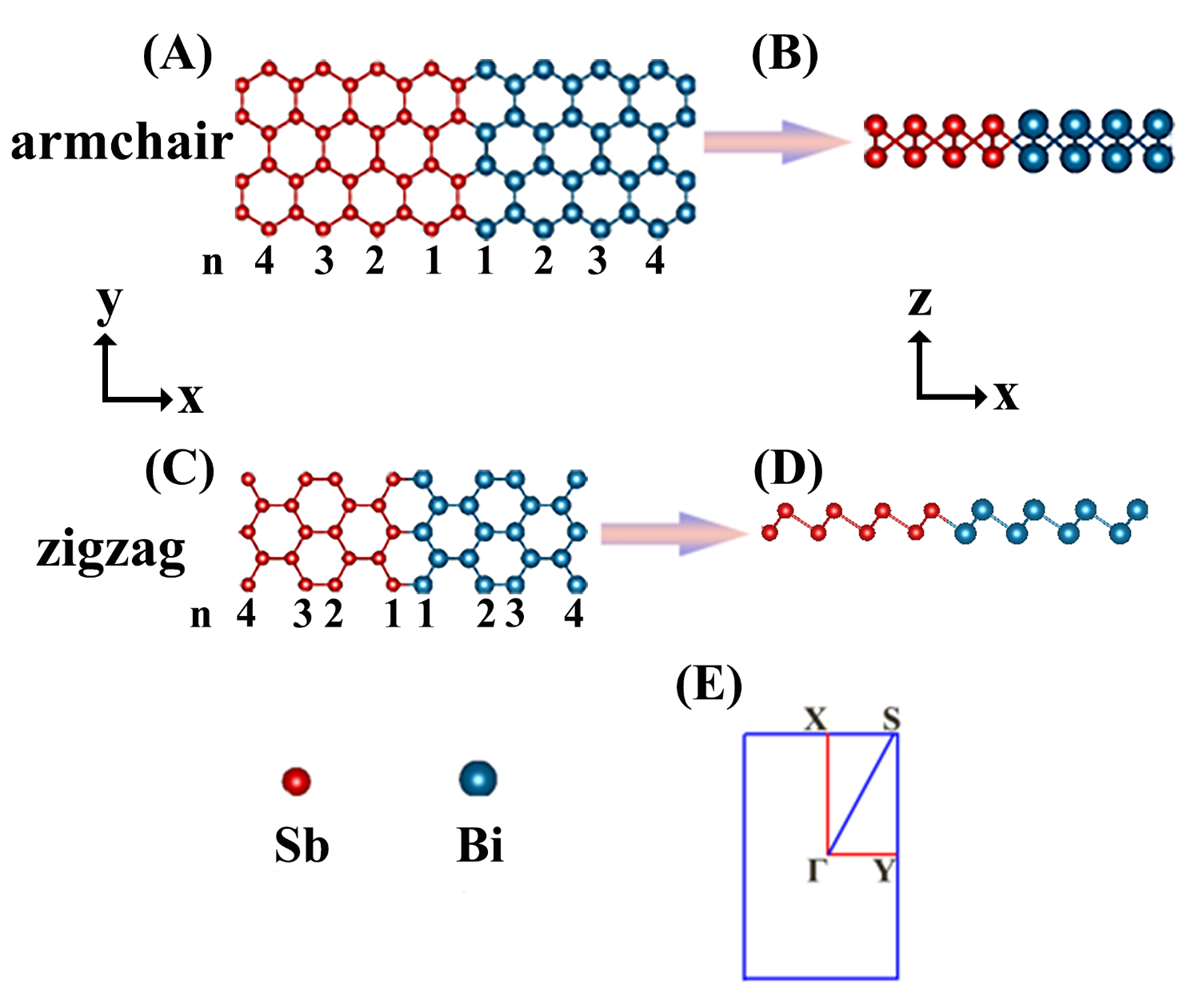


**Supplementary Figure 1.** **(A)** Top view of the armchair Sb_4_/Bi_4_ LHS structure; **(B)** Side view of the armchair Sb_4_/Bi_4_ LHS structure; **(C)** Top view of the zigzag Sb_4_/Bi_4_ LHS structure; **(D)** Side view of the zigzag Sb/Bi LHS structure; **(E)** Brillouin area of the designed structure.


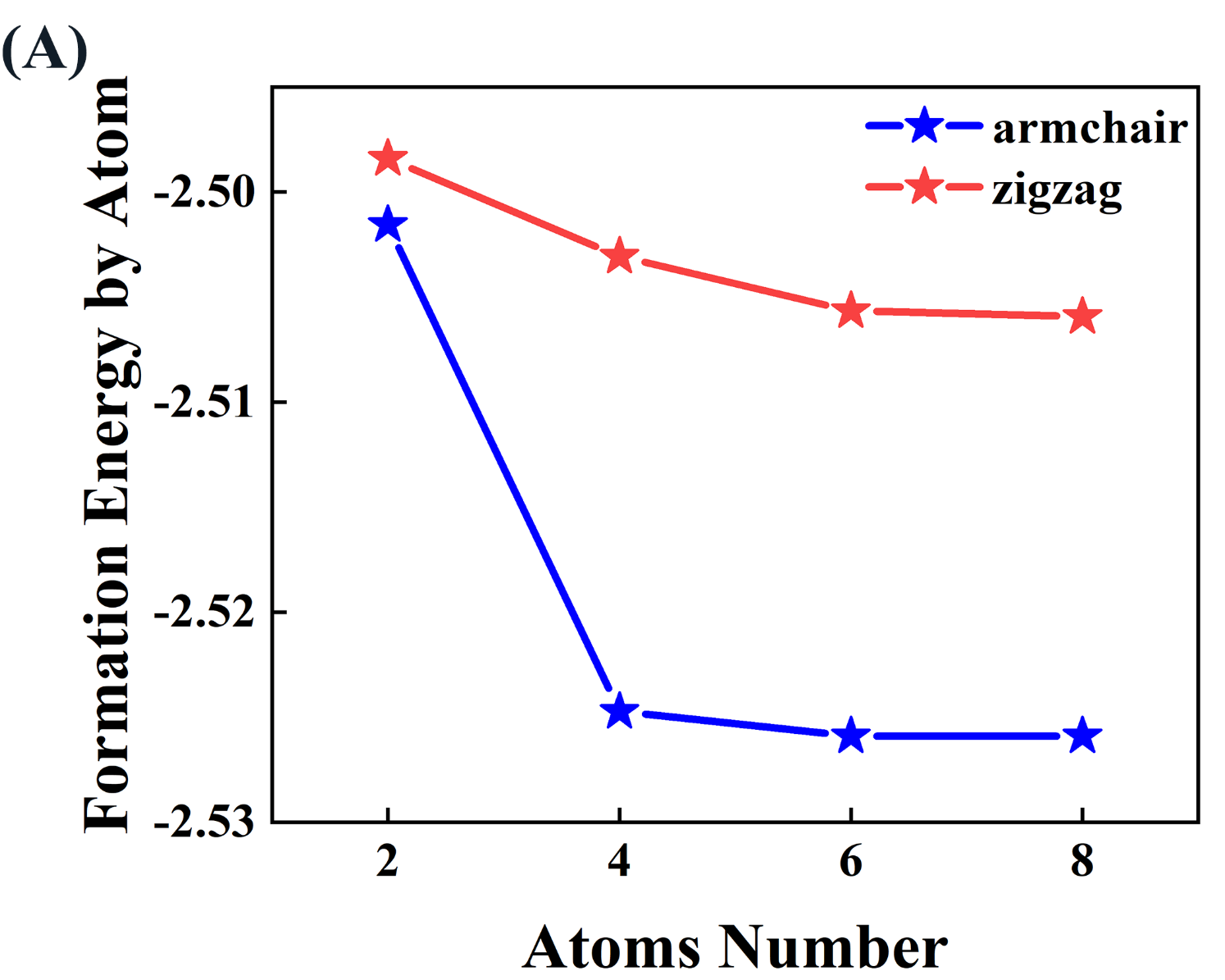


**Supplementary Figure 2.** **(A)**The formation energy by atom of the armchair Sb/Bi LHSs and zigzag Sb/Bi LHSs of the different n.


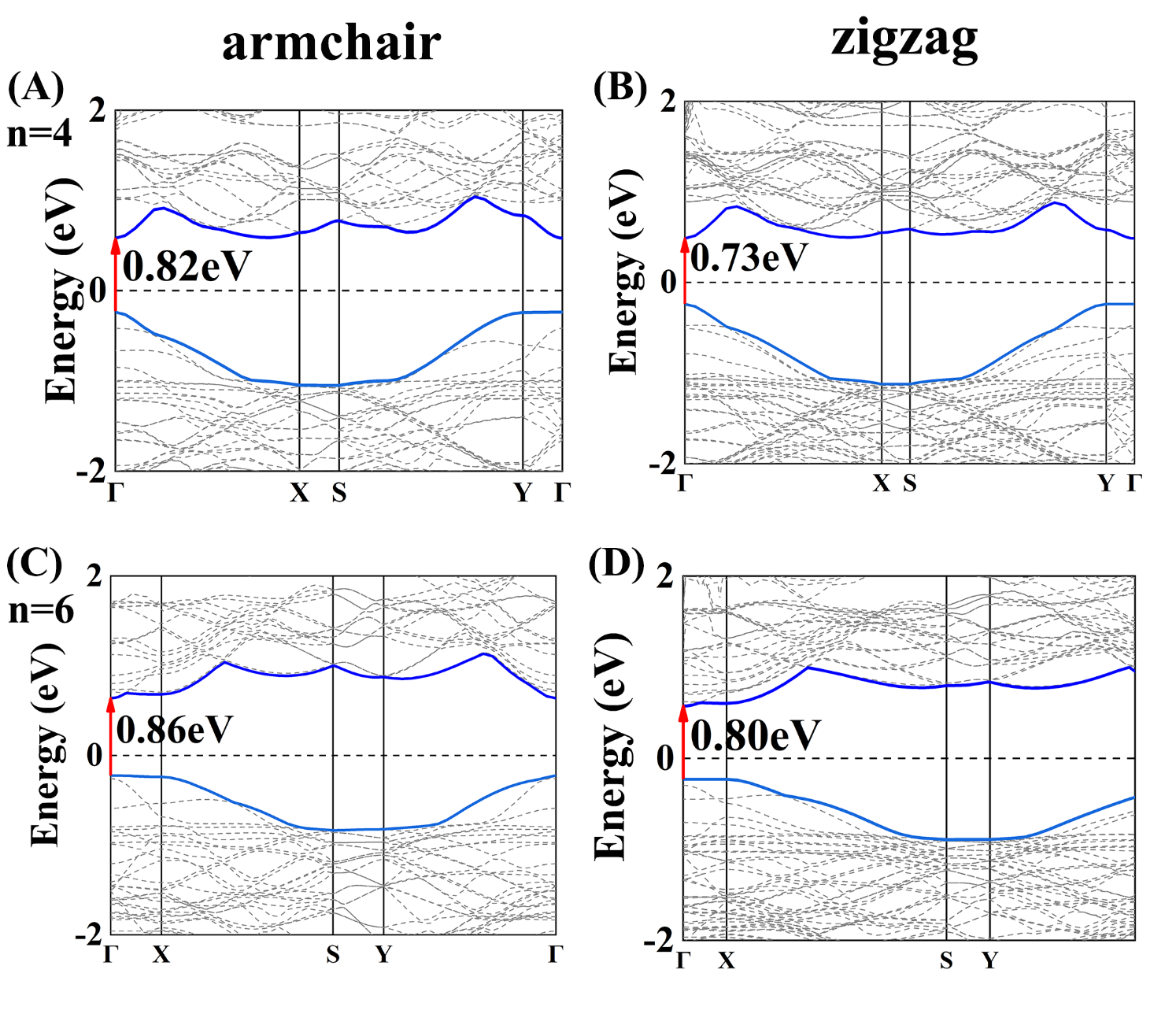


**Supplementary Figure 3.** **(A), (C)** Band structure of the armchair of Sb_4_/Bi_4_ LHSs and Sb_6_/Bi_6_ LHSs, respectively; **(B), (D)** Band structure of the zigzag of Sb_4_/Bi_4_ LHSs and Sb_6_/Bi_6_ LHSs, respectively.

**
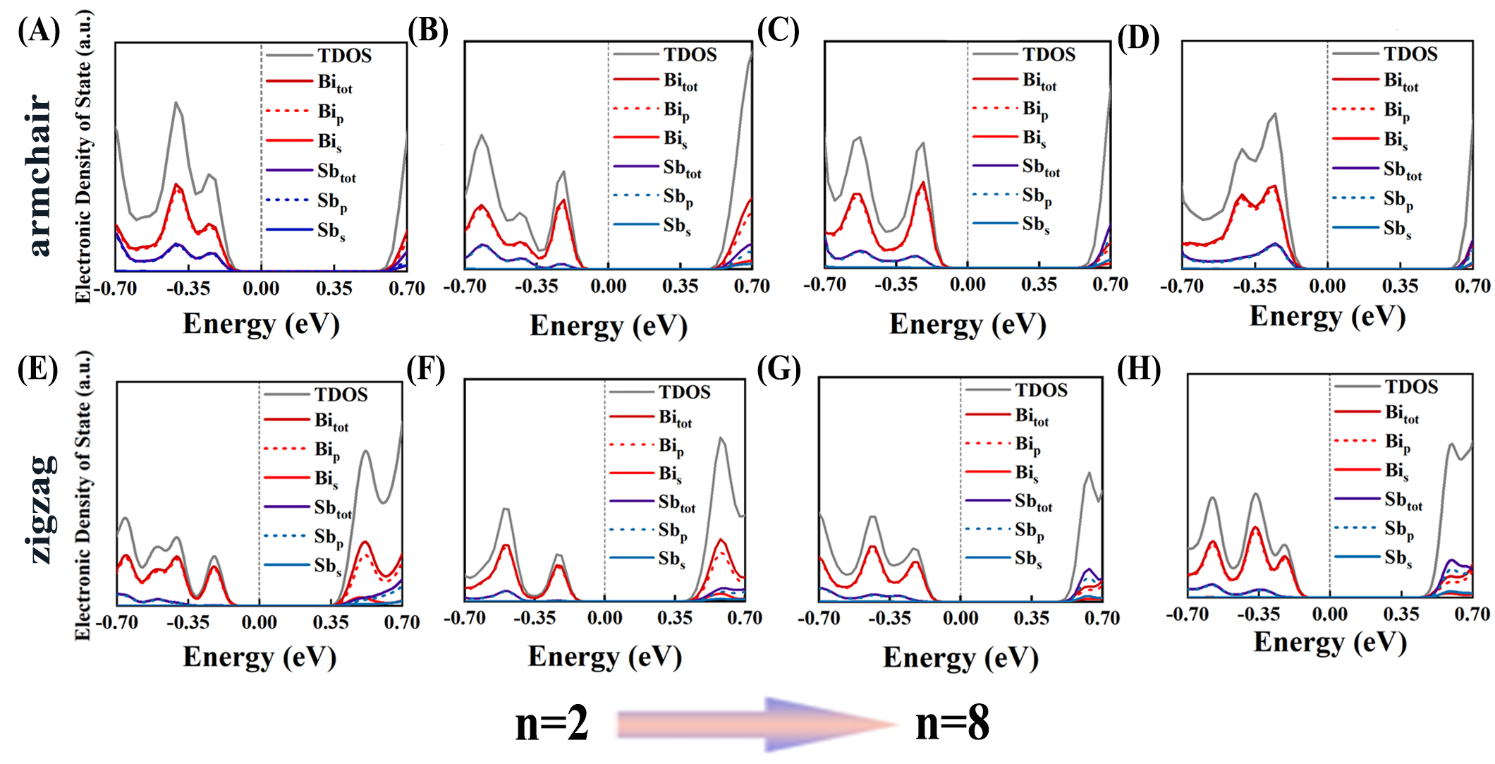
**

**Supplementary Figure 4.** **(A), (B),(C) and (D)** TDOS and PDOS of the armchair Sb_n_/Bi_n_ LHSs under n from 2 to 8, respectively; **(E), (F),(G) and (H)** TDOS and PDOS of states (PDOS) of the zigzag Sb_n_/Bi_n_ LHSs under n from 2 to 8, respectively.

**
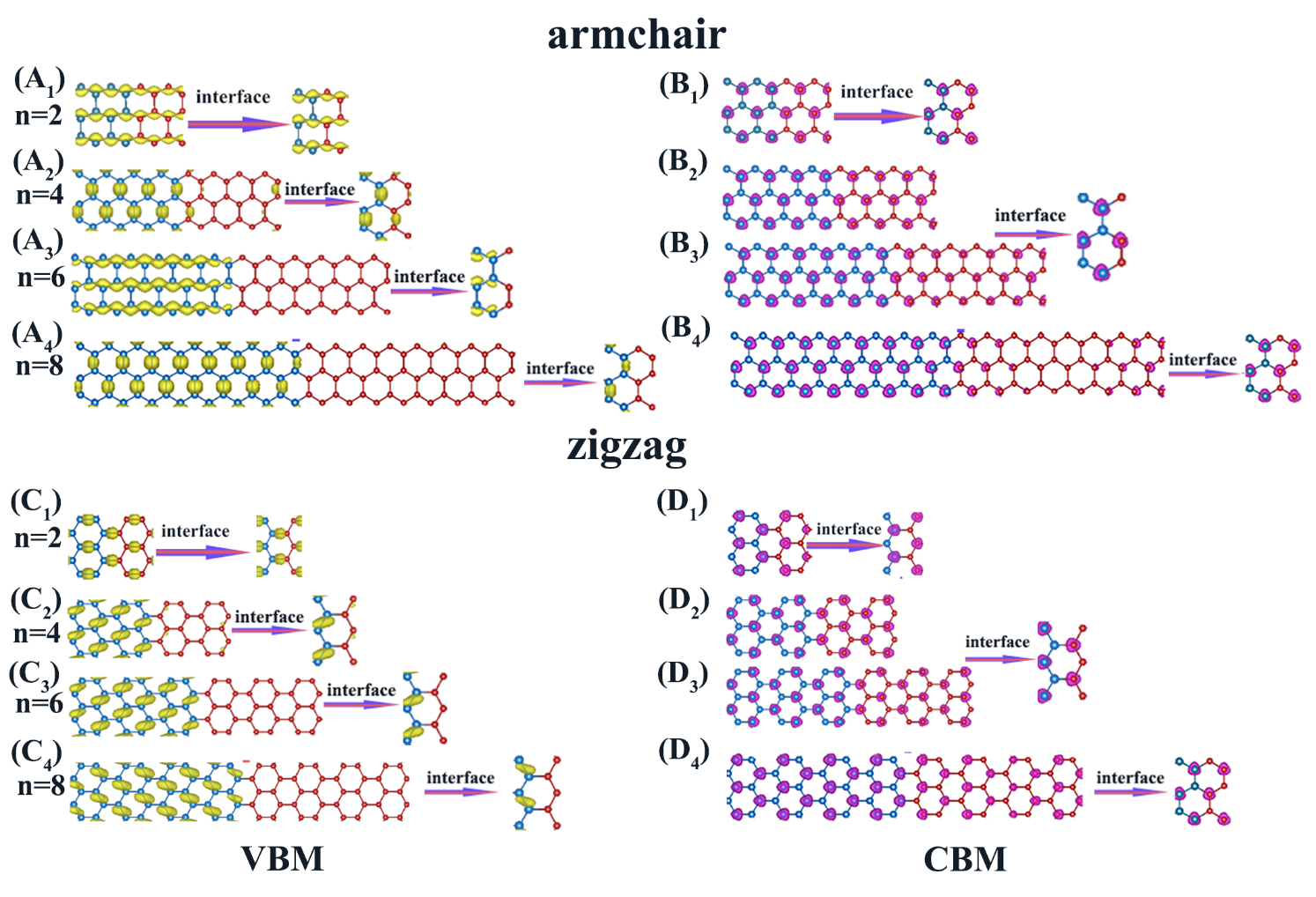
**

**Supplementary Figure 5.** **(A_1_), (A_2_),(A_3_) and (A_4_)** The charges on VBM of the armchair Sb_n_/Bi_n_ LHSs under n from 2 to 8, respectively; **(B_1_), (B_2_),(B_3_) and (B_4_)** the charges on CBM of the armchair Sb_n_/Bi_n_ LHSs under n from 2 to 8, respectively; **(C_1_), (C_2_),(C_3_) and (C_4_)** the charges on VBM of the zigzag Sb_n_/Bi_n_ LHSs under n from 2 to 8, respectively; **(C_1_), (C_2_),(C_3_) and (C_4_)** the charges on CBM of the zigzag Sb_n_/Bi_n_ LHSs under n from 2 to 8, respectively.
